# Valorization of sugarcane bagasse for sugar extraction and residue as an adsorbent for pollutant removal

**DOI:** 10.3389/fbioe.2022.893941

**Published:** 2022-08-24

**Authors:** Duanhao Wang, Jiahua Tian, Jian Guan, Yiwen Ding, Ming Li Wang, Brandon Tonnis, Jiayang Liu, Qingguo Huang

**Affiliations:** ^1^ College of Biology and Food Engineering, Huanghuai University, Zhumadian, China; ^2^ College of Environmental Science and Engineering, Nanjing Tech University, Nanjing, China; ^3^ USDA-ARS, Plant Genetic Resources Conservation Unit, Griffin, GA, United States; ^4^ Department of Crop and Soil Sciences, University of Georgia, Griffin, GA, United States

**Keywords:** sugarcane bagasse (SCB), sugar, hydrolysis, adsorbent, dye, heavy meals

## Abstract

Following juice crushing for sugar or bioethanol production from sugarcane, bagasse (SCB) is generated as the main lignocellulosic by-product. This study utilized SCB generated by a hydraulic press as feedstock to evaluate sugar extraction as well as adsorption potential. Total soluble sugar (sucrose, glucose, and fructose) of 0.4 g/g SCB was recovered with H_2_O extraction in this case. Insoluble sugar, that is, cellulose in SCB, was further hydrolyzed into glucose (2%–31%) with cellulase enzyme, generating a new bagasse residue (SCBE). Persulfate pretreatment of SCB slightly enhanced saccharification. Both SCB and SCBE showed great potential as adsorbents with 98% of methylene blue (MB) removed by SCB or SCBE and 75% of Cu^2+^ by SCBE and 80% by SCB in 60 min. The maximum adsorption amount (*q*_m_) was 85.8 mg/g (MB by SCB), 77.5 mg/g (MB by SCBE), 3.4 mg/g (Cu^2+^ by SCB), and 1.2 mg/g (Cu^2+^ by SCBE). The thermodynamics indicated that the adsorption process is spontaneous, endothermic, and more random in nature. The experimental results offer an alternative to better reutilize SCB.

## 1 Introduction

With the aim of global carbon emission reduction, it is important and meaningful that valuable resources/materials be extracted from the existing agro-industrial wastes. For instance, bioenergy materials—bioethanol, biogas, and biohydrogen—have been produced from lignocellulosic biomass wastes (Martínez et al., 2009; Waclawovsky et al., 2010; Konde et al., 2021). In addition, various agro-waste–based materials have been used as biosorbents for efficient removal of organic and inorganic pollutants from wastewater (Bhatnagar et al., 2015; Mo et al., 2018; Ezeonuegbu et al., 2021). By such endeavors two major goals can be achieved—reducing buildup of wastes in the environment as well as acquiring added values from the wastes. Sugarcane, *Saccharum officinarum*, is an important energy crop for bioenergy production as global biofuel demand surges (Papini-Terzi et al., 2009; Waclawovsky et al., 2010). Intensive sugarcane growing countries, for example, Brazil, India, China, and Thailand, contribute to the total production of sugarcane of 1.91 billion tons annually (Aruna et al., 2021). To utilize sugarcane, the soluble sugar-containing juice, serving as a source of chemicals for direct processing or microbial transformation for bioenergy, must be extracted from the plant by physical squeezing/crushing methods, such as using hydraulic pressure (Wang et al., 2014). Wet sugarcane plant contains roughly 36% juice and 64% residue (30% bagasse and 34% straw and leaves), and the latter contains 50% and 10% moisture, respectively (Ferreira-Leitão et al., 2010; Aruna et al., 2021; Agarwal et al., 2022). The large amount of co-produced sugarcane bagasse (SCB), on the one hand, poses great challenge to processing industries and environment, and on the other hand, might be better utilized as a cheap source to produce value-added products, for example, enzymes, reducing sugars, prebiotic, organic acids, and biofuels (Alokika et al., 2021). For this reason, much attention has been paid in finding effective ways to reuse SCB (Chen et al., 2017).

Despite using high pressure to squeeze juice from sugarcane, sugar is inevitably left behind in the SCB. These residual soluble sugars represent a significant amount and should not be overlooked, which has not been examined in some of the related literature (Chourasia et al., 2021). For instance, bulk SCB collected from “sugarcane juice” vendors was only washed a few times to remove the surface impurities and then dried, ground, and sieved for further use (Kerrou et al., 2021), possibly leaving behind additional sugars inside the biomass. Apart from soluble sugars, another problem associated with utilization of SCB is the less efficient and economical release of compositional sugars from lignocelluloses for biofuel generation *via* enzymatic hydrolysis with cellulase enzymes (Martínez et al., 2009; Waclawovsky et al., 2010; Liu et al., 2015). A variety of pretreatment methods, usually chemical and physicochemical, have been developed to enhance the enzymatic saccharification of cellulose (Tana et al., 2016). Persulfate is an oxidizing reagent with great potential in degrading organic pollutants (Miklos et al., 2018). It is interesting to examine the effect of persulfate pretreatment on saccharification of agro-wastes, which has not been previously investigated.

Rising environmental contamination has put human health at great risk, especially in water bodies that are contaminated with various pollutants—antibiotics (Lyu et al., 2020), dyes (Aruna et al., 2021), pesticides (Gaur et al., 2018), heavy metals (Luo et al., 2021), etc. Even though industrial wastewater is intensively treated prior to discharge, organic pollutants such as dyes are still detected as micropollutants in different environment matrices, such as in water, suspended particulate matters, sediments, and wild fishes (Tkaczyk et al., 2020). Meanwhile, heavy metal ions can enter into the environment, causing negative impacts on ecosystems and human health (Song & Li, 2015; Shahid et al., 2017). The adsorption technique has been proved to be an effective and easy-to-implement approach to clarify wastewater, hence screening or synthesizing cost-effective materials as adsorbents is of great practical importance (Azari et al., 2020). Employing agricultural wastes as adsorbents can possibly achieve dual benefits in terms of solid waste reduction as well as environmental protection (Zhou et al., 2015; Mo et al., 2018; Li W. et al., 2019). Employing original or modified SCB as adsorbent for pollutant removal has been widely reported (Aruna et al., 2021; Ezeonuegbu et al., 2021). However, to our knowledge, to this date, few research works have been performed on evaluation of enzymatically modified SCB as adsorbent for dye and heavy metal removal from water.

The objectives of this study were to utilize hydraulic press–generated SCB as starting feedstock to assess 1) extraction of the residual soluble sugars in initial SCB, 2) enzymatic hydrolysis of SCB from the aforementioned step with different pretreatments, and 3) adsorptive removal of pollutants from water using SCB and SCBE after enzymatic hydrolysis. This study reports an integrative approach of how to valorize SCB—extract sugars for potential bioenergy production and further use the generated residue as adsorbent for pollutant removal.

## 2 Materials and methods

### 2.1 Bagasse biomass

Sugarcane seeds (PI: HoCP04-838, LCP-384, LCP-384) were planted in the screen house at the early spring of 2013 in the Griffin campus of University of Georgia, United States. The plants were harvested at physiological maturity (Wang et al., 2014). The leaves were removed and fresh stems were hydraulically pressed for juice collection and quantification of sugar concentration (Wang et al., 2014). After pressing for juice study in the late autumn in 2013, the residual bagasse was also collected and instantly dried using a forced-air oven at 80°C to a constant weight. The dried bagasse was used as feedstock in this study. The dried bulk sugarcane bagasse (SCB) was combined, ground, and passed through a 2 mm mesh and then stored at room temperature until use.

### 2.2 Chemicals and cellulase enzyme

Sugar standards were procured from Sigma-Aldrich Chemicals, United States. Potassium persulfate (catalog number 105091) was purchased from Merck. Deionized (DI) water was used for dissolving chemicals and sugar extraction. Liquid cellulase enzyme blend (Cellic CTec2, Lot# SLBS6227), a product of Novozyme Corp., was purchased from Sigma. The enzyme blend containing cellulases, β-glucosidases, and hemicellulases was directly used for hydrolysis without any further treatment or dilution. Cellulase activity in filter paper unit (FPU) was determined as previously described (Liu et al., 2013). A syringeless filter device (Mini-UniPrep™, 0.45 μm pore size) from Whatman™ (GE Healthcare UK Limited.) was used to prepare samples for high-performance liquid chromatography (HPLC).

### 2.3 Soluble sugar extraction from the original bagasse

Soluble sugar extraction from the original SCB was performed in a 200-ml flask in which 1 g of SCB on a dry weight basis and 20 ml DI water were added. The flasks were sealed with parafilm and put on a rotatory shaker for 2 h at 150 rpm and room temperature. Subsequently, the sugar-containing liquid was separated from the solid bagasse using vacuum filtration. The bagasse was further extracted second and third time using the same procedure. The liquid sample from each batch of extraction was analyzed for sugar content, and the sum of these was used to calculate the total sugar recovery from the bagasse. The sugars in the second and third batch extractions only contributed less than 5% of the total sugars, and therefore the efficiency of the first batch extraction was further studied by varying the DI water dose (20 ml) and extraction time (2 h). Each extraction experiment was done in triplicate, and the mean values of the results (SD ≤ 5%) were used in plotting the stacked bar figures.

### 2.4 Enzymatic hydrolysis of soluble sugar–free sugarcane bagasse

Following the aforementioned operation, residual soluble sugars were assumed to be fully extracted, leaving soluble sugar–free SCB, containing mainly lignocellulose. The insoluble sugar, that is, cellulose and hemicellulose, was further digested with a commercial cellulase-containing enzyme cocktail to release structural sugars, such as glucose. Enzymatic hydrolysis of SCB was carried out according to the methods described by the National Renewable Energy Laboratory (NREL/TP-500-42629) with some modifications (Liu et al., 2013). To each 20-ml glass reactor, 0.1 g of SCB on a dry mass basis, enzyme solutions in different volumes (0.01, 0.05, 0.1, 0.3, 0.5, 0.7, and 1 ml), 400 μg tetracycline, and 300 μg cycloheximide were added. Then 0.1 M citrate–phosphate buffer (pH 4.8) was added to bring the final volume to 10 ml in each reactor. The vials were tightly capped and incubated at 50°C for 24 h with periodic manual shaking to hydrolyze the bagasse. Finally, the liquid and solid in each reactor was separated by vacuum filtration. The liquid taken from each reactor was sampled for sugar content analysis by using Agilent 1100 HPLC with an autosampler and a refractive index detector (RID) as described later, while the solid residue of enzyme-digested SCB (referred as SCBE) was washed with deionized water three times to remove the attached chemicals. SCB and SCBE were dried using a forced-air oven at 80°C to a constant weight before further use and analysis.

### 2.5 Effect of persulfate pretreatment of sugarcane bagasse or laccase addition on enzymatic hydrolysis

The soluble sugar-free SCB prepared in Section 2.3 was also tested by pretreatment with potassium persulfate prior to enzyme hydrolysis. Each experiment was performed three times with the mean values ± SD of the results used in plotting. The pretreatment was performed by adding SCB into the solution of potassium persulfate (PS) at the final ratio varying from 0.1 to 2 g PS/g SCB for 1 h. After the pretreatment, the solid bagasse was separated from the solution by vacuum filtration. Enzymatic hydrolysis was then conducted at a higher cellulase dosage (∼38.6 FPU) per gram of persulfate-treated SCB. Since the persulfate pretreatment at the 0.1 g/g SCB dosage was effective in improving hydrolysis, further test was performed at this dosage with ferrous sulfate at the dosage of 0.02–0.1 g/g SCB added with the persulfate to test its synergistic effect on hydrolysis. In all tests, the persulfate-pretreated SCB was carefully collected and oven dried at 80°C for 24 h prior to the enzyme hydrolysis treatment. For enzyme hydrolysis treatment, additional tests were conducted by adding 10 or 50 U laccase/g SCB to the enzymatic solution for verifying its effect on hydrolysis efficiency. The laccase enzyme was homemade with a fungal strain and its activity was determined with substrate DMP (ε= 49.5 mM^−1^ cm^−1^), as previously described (Liu et al., 2016). The increase in absorption at 470 nm was recorded in a reaction system of 3 ml that comprised 2.4 ml citrate–phosphate buffer (20 mM, pH = 3), 0.5 ml DMP solution (10 mM), and 0.1 ml enzyme solution. One unit of laccase activity was equal to the amount of enzyme that oxidized 1 μmol of DMP per min.

### 2.6 Sugar quantification with high-performance liquid chromatography

HPLC (Agilent 1100 liquid chromatography with a refractive index detector) was used to quantify sugars according to the standard methods (NREL/TP-500-42618) and following a method described in our earlier study (Wang et al., 2014). Sugar standards (i.e., fructose, glucose, sucrose, xylose, mannose, galactose, and arabinose) were dissolved and diluted in deionized (DI) water to the following concentrations (mg/ml): 0.1, 0.2, 0.5, 1.0, 2.0, and 4.0. The generated standard curves were used for peak identification and quantification. The extraction solution or enzymatic hydrolysate was neutralized to around pH 7.0 using an appropriate amount of sodium bicarbonate and then filtered through a 0.45-μm PVDF filter membrane prior to the HPLC analysis (Liu et al., 2013). The glucose concentration (265.6 mg/L) in the cellulase product was also determined with HPLC, which was accordingly subtracted from the results of the sugar concentration in the different hydrolysates following enzymatic hydrolysis experiments.

### 2.7 Physical and chemical characterization of sugarcane bagasse and SCBE

For the following adsorption study, SCBE was prepared from hydrolysis with a low cellulase enzyme dosage of around 3 U/g bagasse without any persulfate or laccase pretreatment. The SCB and SCBE were analyzed in terms of chemical composition, including lignin, cellulose, hemicellulose, and ash (Koutrotsios et al., 2014). The physical properties such as specific surface area (SSA), total pore volume (TV), and average pore size (APS) were determined using the nitrogen adsorption–desorption isotherm curve with a Brunauer–Emmett–Teller (BET) surface area and a micropore size analyzer JW-BK100A (JWGB Sci. & Tech. Com., Ltd., China). FT-IR spectra of bagasse in the range of 4000–400 cm^−1^ was recorded using a Nicolet iS10 Fourier transform infrared (FTIR) spectrometer (Thermo Fisher Scientific, USA). The dried SCB or SCBE was mixed with KBr at 1:100 g/g, grinded well in a mortar, and subjected to a tablet pressing machine to make a thin sheet before scanning. The collected scanning data were directly used for plotting.

### 2.9 Adsorption of methylene blue dye and Cu^2+^ metal with sugarcane bagasse and SCBE

Methylene blue (MB) or metal ion adsorption was carried out in a 250-ml flask containing 50 ml solution at different pollutant concentration (MB: 10–400 mg/L; Cu^2+^: 1–80 mg/L), contact time (MB: 0–12 h; Cu^2+^: 0–4 h), and temperature (MB: 25–55°C; Cu^2+^: 30–50°C) with 2 g/L dried adsorbent (SCB or SCBE). The pH value of the adsorption system was 7.5–7.69 for MB ranging from 10–400 mg/L and adjusted to 4 with 0.1 M HNO_3_ for Cu^2+^ ranging from 1–80 mg/L for better adsorption performance, as suggested in previous reports (Homagai et al., 2010). Each adsorption experiment was conducted in triplicate under one variable, while others were fixed and the mean value of the results was used to plot (SD ≤ 5%). After adsorption, the solution in each flask was centrifuged at 4000 rpm for 10 min to obtain the supernatant in which residual pollutant concentration was determined (Han et al., 2010). The pollutant removal rate (Eq. 1) and adsorption amount at equilibrium (*q*_e_) (Eq. 2) were calculated as follows:

$$\text{R}=\frac{c_{0}-c_{\text{e}}}{c_{0}}\times 100\text{\%,}$$ (1)

$$q_{\text{e}}=\frac{\left(c_{0-}c_{\text{e}}\right)\times \text{V}}{\text{W}},$$ (2)

where *c*_o_ is initial pollutant concentration (mg/L); *c*_e_ is equilibrium dye concentration (mg/L); V is volume of dye solution (L); W is weight of adsorbent (g); and *q*_e_ is equilibrium adsorption amount (mg/g). All adsorption experiments were performed in triplicate with relative standard deviations < 5%, and the averages for triplicate data were reported in the results.

The adsorption data were then fitted with Lagergren’s pseudo-first-order model (Eq. 3) and Ho’s pseudo-second-order model (Eq. 4).

$$\mathrm{lg}\left(q_{e}-q_{t}\right)=-\frac{K_{1}t}{2.303}+\mathrm{lg}q_{e},$$ (3)

$$\frac{t}{q_{t}}=\frac{t}{q_{e}}+\frac{1}{K_{2}q_{e}^{2}},$$ (4)

where *q*_e_ is equilibrium dye adsorption of the adsorbent (mg/g); *q*_t_ is the adsorbed amount of adsorbent (mg/g) at contact time t (h or min); *K*_1_ is the equilibrium rate constant of the first-order sorption (min^−1^); and *K*_2_ is the equilibrium rate constant of the second-order sorption (g/mg·min).

The Langmuir (Eq. 5) and Freundlich (Eq. 6) equations were employed to explicate the sorption isotherms of MB or Cu^2+^ on the bagasse.

$$\frac{C_{e}}{q_{e}}=\frac{1}{K_{L}q_{m}}+\frac{C_{e}}{q_{m}},$$ (5)

$$\mathrm{lg}q_{e}=\frac{1}{n}\mathrm{lg}c_{e}+\mathrm{lg}K_{F},$$ (6)

where *K*_*L*_ (L/mg) is the Langmuir adsorption constant, *q*_m_ (mg/g) is the maximum dye amount of adsorption corresponding to complete monolayer coverage on the surface, *q*_*e*_ (mg/g) is the amount of dye adsorbed by sorbent at equilibrium, *c*_e_ (mg/L) is the equilibrium concentration of dye solution, and *K*_*F*_ is an indicator of adsorption capacity (mg/g) and 1*/n* is the adsorption intensity.

The change in free energy (Δ*G* ^o^) was evaluated using the Eq. 7 to study the thermodynamic nature.

$$\Delta G^{{}^{\circ}}=-RT\ln\left(\frac{q_{e}}{c_{e}}\right),$$ (7)

where R is gas constant (8.3143 J mol^−1^ K^−1^); T is absolute temperature (Kelvin); *q*_e_ is equilibrium adsorption amount (mg/g); and *c*_e_ is equilibrium dye concentration (mg/L). From the plot of Δ*G* ^o^ vs. T, the value of enthalpy Δ*H* ^o^ and entropy Δ*S* ^o^ was calculated as follows:

$$\Delta G^{{}^{\circ}}=\Delta H^{{}^{\circ}}-T\Delta S^{{}^{\circ}}.$$ (8)

## 3 Results and discussion

### 3.1 Sugar extraction with water and enzymatic hydrolysis

**FIGURE 1:**
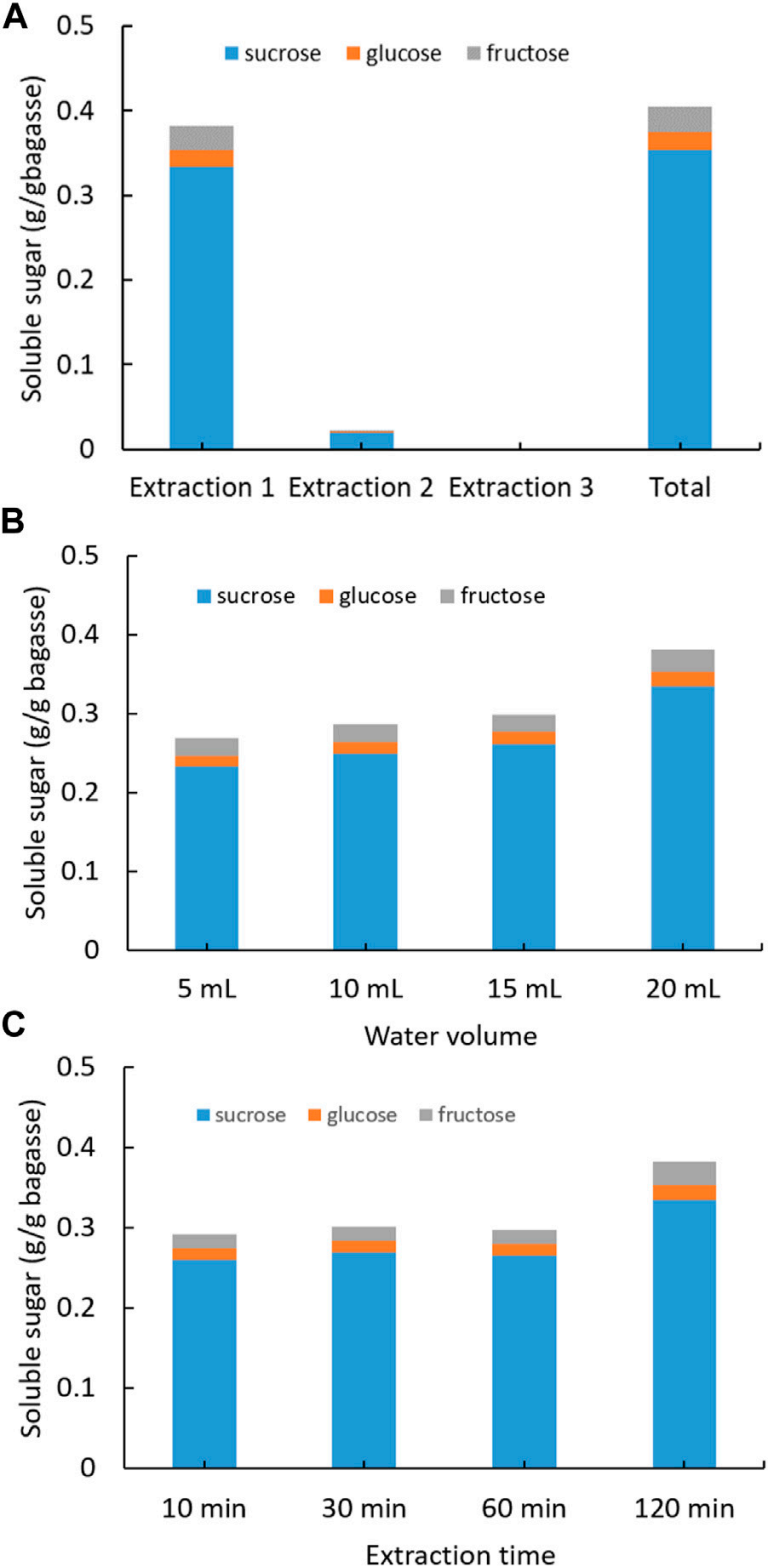
Extraction of soluble sugars from SCB with water under different conditions: **(A)** three consecutive extractions, **(B)** varying water volume, and **(C)** varying extraction time. (Standard deviation ≤ 5% for sucrose, glucose, and fructose).

The initial SCB, which was hydraulic press generated from sugarcane stems and oven dried at 80°C, was soaked into DI water to extract soluble sugars (Figure 1). Three consecutive extractions were conducted for each SCB sample in which sugar concentration was quantified with HPLC. The first extraction yielded the highest recovery of sugars, that is, 0.33 g sucrose, 0.01 g glucose, and 0.03 g fructose from 1 g of SCB (Figure 1A). The following second and third extractions produced very low sugar recovery. The total sugars recovered from three extractions added up to 0.41 g from 1 g of dried SCB. In a previous report, fresh juice from sugarcane stems obtained using hydraulic press was found to contain sucrose 90.5 mg/ml, glucose 6.9 mg/ml, and fructose 4.9 mg/ml (Wang et al., 2014), some of which were inevitably retained in the dried SCB. To maximize the extraction efficiency, different amount of water and extraction time on sugar recovery was also tested (Figures 1B,C). It was observed that with 20 ml of water and duration of 2 h, the highest sugar extraction rate was reached. The results demonstrate that there could be considerable amounts of leftover sugars in the bagasse SCB following juice squeezing, and these sugars can be easily recovered by using water soaking and solid–liquid separation. The highest content of extracted sugars from bagasse was identified as sucrose, which is in agreement with that in mature internodes of the plant culm (Papini-Terzi et al., 2009; Benjamin et al., 2014). It is noteworthy that the residual sugar content might vary greatly among bagasse of different sugarcane plant cultivars and squeezing approaches.

**FIGURE 2:**
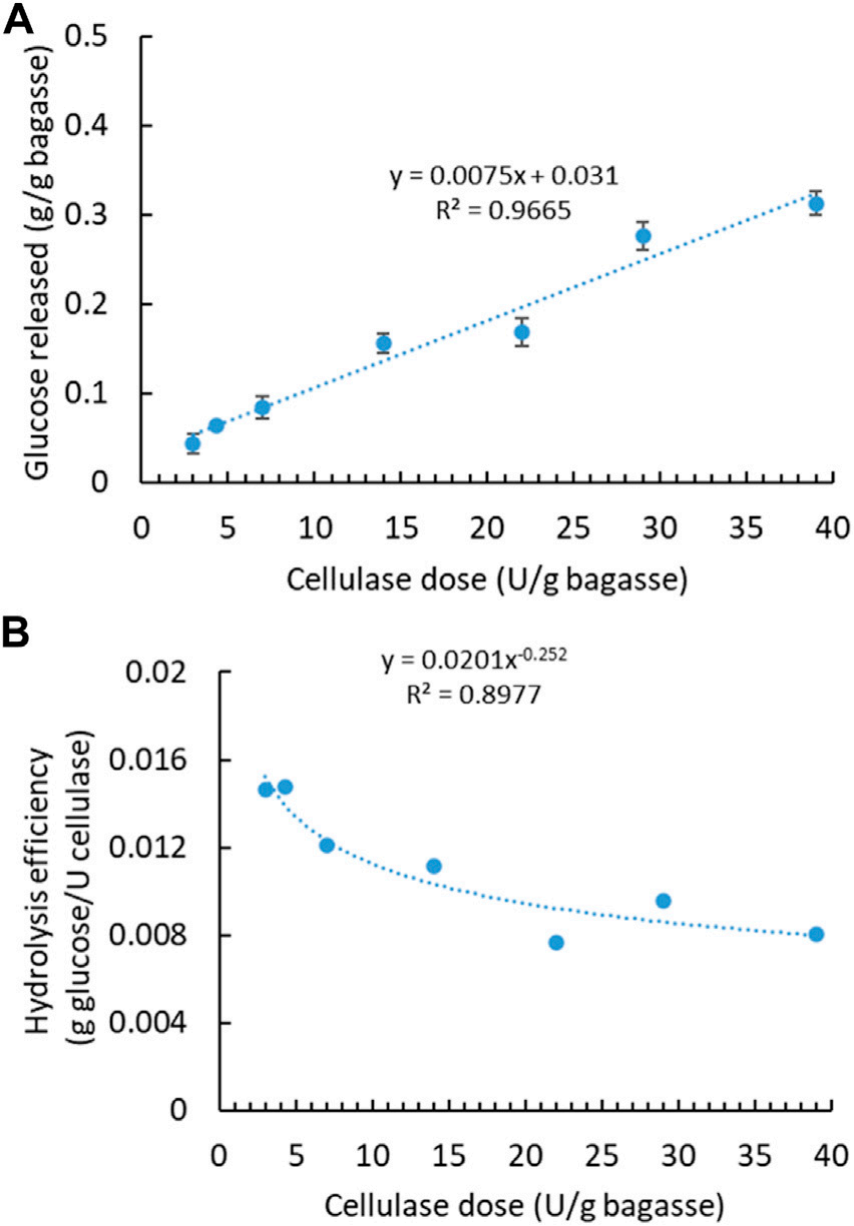
Enzymatic hydrolysis of soluble sugar-free SCB with cellulase enzyme. **(A)** Effect of cellulase dosage on glucose concentration. **(B)** Hydrolysis efficiency versus cellulase dosage. (Reaction condition: 0.1 g of SCB, varying cellulase dosages, 400 μg tetracycline and 300 μg cycloheximide, pH 4.8, temperature 50°C, and time 24 h. Final volume: 10 ml in each vial).

With aforementioned water extraction, the initial SCB was regarded soluble sugar-free SCB, mainly containing lignocellulose—lignin, cellulose, and hemicellulose. To maximize the sugar production, soluble sugar-free SCB was further digested with cellulase to release fermentable monosaccharides from structural polysaccharides. Cellulase had been applied at varying dosages, and the released sugar content was quantified accordingly (Figure 2A). Glucose was found to be the most predominant one among monosaccharides, while others were detected at very low concentrations, therefore not shown in the following figures. Along with the increasing enzyme dosage, more glucose was generated with the highest concentration of 0.31 g from 1 g of SCB at the highest enzyme dosage. There is a linear correlation between the released sugar content and the applied enzyme dosage. Variation in enzymatic hydrolysis, that is, glucose content of 0.17–0.39 g/bagasse, has been observed in different sugarcane varieties in two harvest years and with dilute acid pretreatment (Benjamin et al., 2014). In light of enzyme cost, it is not always the most economical or efficient to use the highest enzyme dosage. Therefore, hydrolysis efficiency (g glucose/U cellulase) versus enzyme dosage is plotted in Figure 2B. Hydrolysis efficiency dropped drastically when a higher enzyme dosage was applied. A lower enzyme dosage led to relatively higher hydrolysis efficiency even though less total sugar was released. Comparatively, sugar (i.e., glucose) recovery by enzymatic hydrolysis is more difficult than soluble sugar extraction from the original SCB using water.

### 3.2 Effect of persulfate pretreatment or laccase addition on enzymatic hydrolysis

**FIGURE 3:**
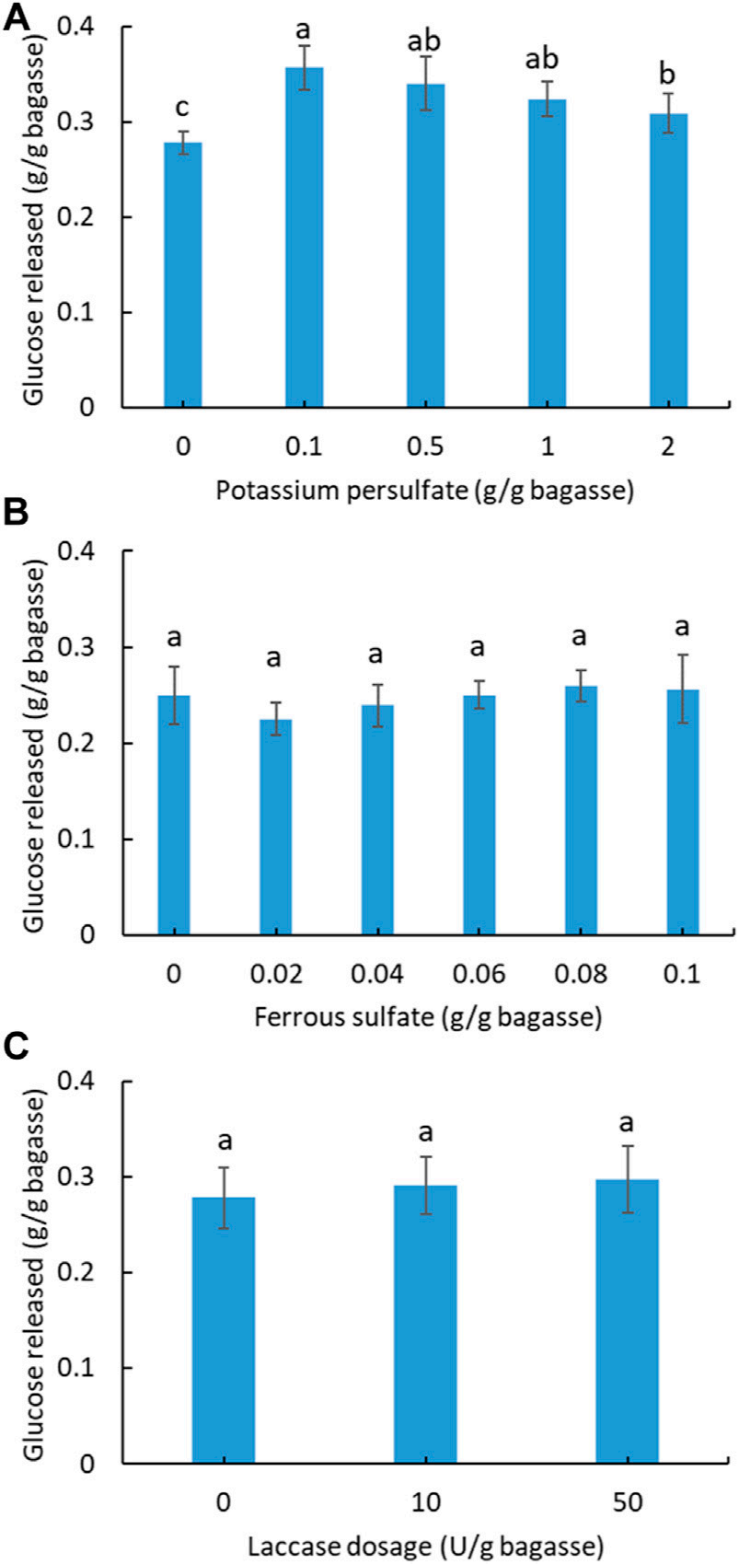
Effect of potassium persulfate **(A)** and activated persulfate with ferrous sulfate pretreatment **(B)** of SCB on the subsequent hydrolysis. **(C)** Effect of laccase addition on the hydrolysis of SCB. (Reaction condition: 0.1 g of pretreated SCB, cellulase ∼38.5 U/g bagasse, 400 μg tetracycline and 300 μg cycloheximide, pH 4.8, temperature 50°C, and time 24 h. Final volume: 10 ml in each vial.) (Glucose content within all columns with a letter in common are not significantly different at *p* < 0.05 level, *n* = 3.)

In order to improve the enzymatic hydrolysis, pretreatment of soluble sugar-free SCB with potassium persulfate was investigated (Figure 3A). A total of four gradients of pretreatment were applied, from which the best enhancement in hydrolysis was found using 0.1 g persulfate/g SCB. The other three dosages also exhibited a positive effect compared with the unpretreated SCB. Based on the best pretreatment condition (0.1 g persulfate/g SCB), ferrous sulfate was further added into the pretreatment system to test its synergistic effect on hydrolysis *via* the sulfate radical advanced oxidation processes (SR-AOPs) (Xu et al., 2021). As shown in Figure 3B, no significant increase in hydrolysis was observed for the applied dosage of ferrous sulfate. By contrast, a small inhibitory effect on hydrolysis was observed in the pretreatment with ferrous sulfate.

Laccase is known as a lignin-degrading enzyme with the potential to assist cellulase digestion of lignocellulose (Giacobbe et al., 2018; Raj & Krishnan, 2018). In the case of SCB in this study, laccase addition at two different dosages did not improve hydrolysis (Figure 3C), which contrasts with a previous study showing that treatment of SCB with a cellulase and laccase (200 U/g) mixture increased the sugar yield by 20% compared to that without laccase (Raj & Krishnan, 2018). This might be ascribed to the lower laccase dosage (≤50 U) applied in this study.

### 3.3 Adsorption of methylene blue and Cu^2+^ with sugarcane bagasse and SCBE-influencing factors

**FIGURE 4:**
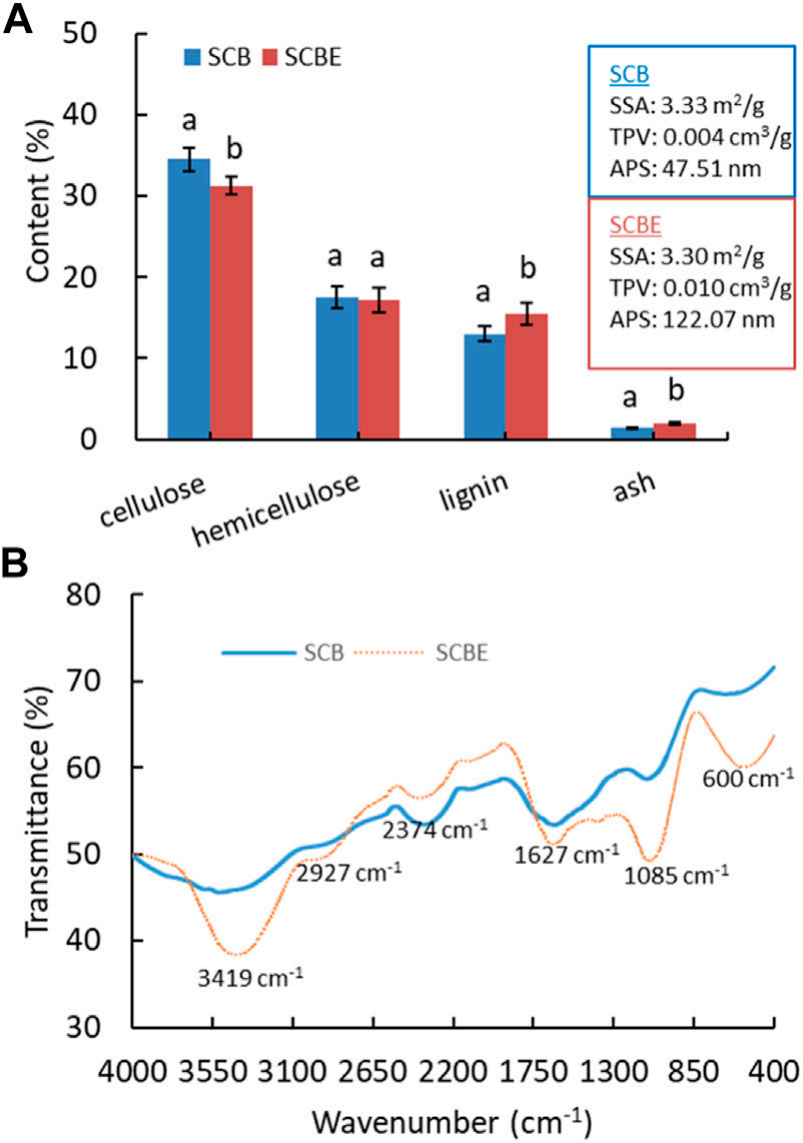
Chemical composition **(A)** and FTIR spectra **(B)** of SCB and SCBE. Hydrolysis was carried out with a low cellulase dosage of 3 U/g bagasse.

Following soluble sugar extraction and enzymatic hydrolysis, the derived SCB and SCBE were then repurposed as adsorbents. It is known that the adsorption efficiency/capacity largely depends on surface morphology as well as physicochemical properties. Therefore, SCB and SCBE were first characterized for chemical composition, physical properties, and surface functional groups (Figure 4). The content of cellulose, hemicellulose, lignin, and ash in SCB was determined as approximately 34.6%, 17.4%, 13.2%, and 1.4%, which was shifted to 31.2%, 17.2%, 15.6%, and 2.0% in SCBE, respectively. Generally, the chemical composition of SCB in this case was similar to but a little different from other reports (Homagai et al., 2010; Benjamin et al., 2014; Ezeonuegbu et al., 2021), which might be explained by the variations in sugarcane plant variety, cultivation region, and growing conditions. Enzymatic hydrolysis resulted in the decrease in cellulose and hemicellulose content, and in the meantime the increase in lignin and ash content. After hydrolysis, physical properties of SCB also changed slightly, specifically the total pore volume (TPV) and average pore size (APS), while the specific surface area (SSA) remained mostly unchanged (around 3.3 m^2^/g). Specifically, cellulase hydrolysis led to larger TV (from 0.004 to 0.01 cm^3^/g) and APS (from 47.5 to 122.1 nm) in SCBE, presumably resulting from the breakdown of cellulose chain by cellulase. In previous reports, without water extraction for soluble sugars, SCB was characterized non-porous or macro-porous based on the SSA of 0.73 m^2^/g, TV of 0.0007 cm^3^/g, and APS of 5.73 nm, respectively (Kerrou et al., 2021), which were apparently lower than the values in this study by 5 to 8 folds. It is apparent that prior water extraction of soluble sugars spares more pore space in SCB that is originally taken by sugar molecules. Therefore, it can be concluded that SSA, TV, and APS can be increased in the following order: initial SCB < soluble sugar-free SCB < SCBE after enzymatic hydrolysis.

To enhance adsorption capacity and efficiency, various modification methods have been applied on SCB to change its physiochemical properties and functional groups. Enzymatic hydrolysis can be regarded as a kind of modification of SCB as its functional groups on the surface were significantly altered as shown in FTIR spectra (Figure 4B). In comparison to the original SCB, enzymatically hydrolyzed adsorbent (SCBE) showed the intensified absorption peak at 3419 cm^−1^ (-OH), 2927 cm^−1^ (-CH_2_ asymmetric stretching), 1627 cm^−1^ (-C=O stretching), 1085 cm^−1^ (-C-OH vibration), and 600 cm^−1^, while in the range of 2650 to 1750 cm^−1^, the absorption intensity was notably weakened (Bisla et al., 2020; Kerrou et al., 2021). The band at wavenumber of around 1748 cm^−1^ has been assigned to carbonyl groups (Ezeonuegbu et al., 2021). Lignin is an irregular polyphenolic polymer, and all its phenyl propanoid units—coniferyl alcohol, sinapyl alcohol, and coumaryl alcohol—have at least one -OH group on the benzene ring (Dong et al., 2018). This may partly account for the absorbance increase in the broad peak of 3410 cm^−1^ in SCBE. In addition, the strong absorbance of C-O band at 1051 cm^−1^ further verified the existence of a rich lignin structure in SCBE (Han et al., 2010). Previous studies have established the important role of hydroxyl and carbonyl acidic groups in adsorption of dyes on biomass through hydrogen, electrostatic, chemical, and van der Waals interactions. The adsorption process could be controlled by various mechanisms in a complicated manner because the enriched hydroxyl and carbonyl groups in SCBE did not improve adsorption in this study, discussed later. In other words, the original SCB had more functional groups in a specific region (2650–1750 cm^−1^), which may govern the high adsorption capacity toward MB and Cu^2+^, as shown in the latter section.

**FIGURE 5:**
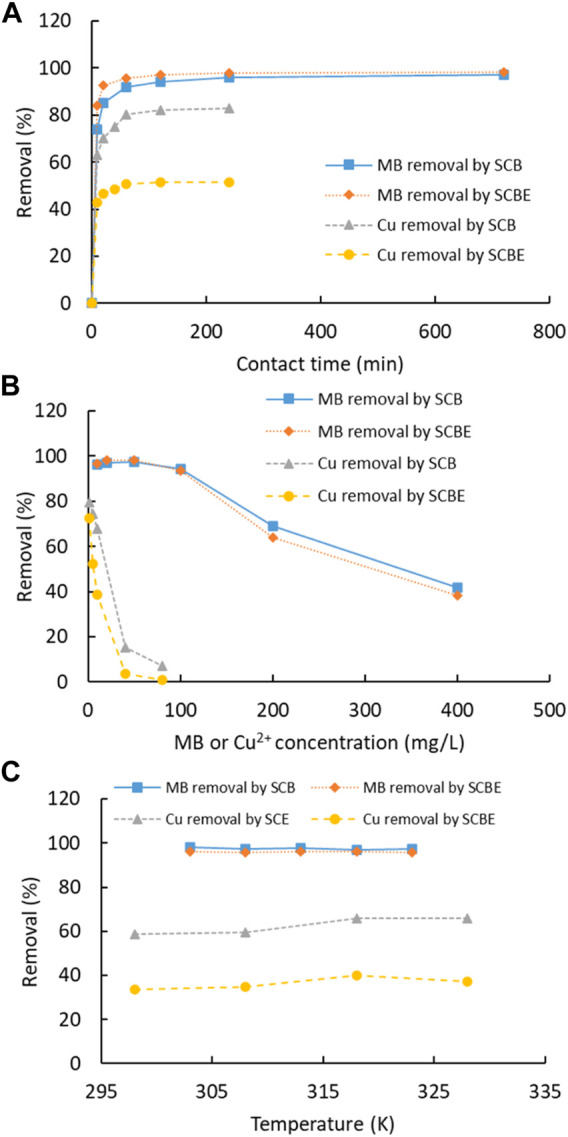
**(A)** Effect of contact time (MB 50 mg/L, Cu^2+^ 10 mg/L, T 25°C, adsorbent 2 g/L, and pH natural for MB and 4 for Cu^2+^), **(B)** initial concentration (T 25°C, adsorbent 2 g/L, time 120 min, and pH natural for MB and 4 for Cu^2+^), and **(C)** temperature (MB 50 mg/L, Cu^2+^ 10 mg/L, adsorbent 2 g/L, time 120 min, and pH natural for MB and 4 for Cu^2+^) on dye MB or Cu^2+^ removal by SCB and SCBE.

Adsorption of dye MB and Cu^2+^ was carried out as a function of contact time, pollutant concentration, and temperature (Figure 5). For both MB and Cu^2+^, the adsorption by the four adsorbents was fast and equilibrium was reached within 60 min. Similarly, another study showed that adsorption of MB on SCB was reached in only 20 min (Kerrou et al., 2021). In comparison of the two adsorbents, the time for adsorption equilibrium by SCBE was slightly shorter than that by SCB, even though SCBE showed reduced adsorption capacity, as revealed in the later section. As for each individual pollutant, the MB removal rate (98%) at equilibrium by SCB and SCBE was almost equivalent, while a reduced removal rate was observed for Cu^2+^ by SCBE (51%) compared to SCB (83%). An elongated time for equilibrium was observed for Cu^2+^ compared with MB in this study, which was similar to previous reports using modified wheat straw as an adsorbent (Han et al., 2010). Along with the increased pollutant concentration, the removal rate descended accordingly, which was more obvious for adsorption of Cu^2+^. At lower concentrations, as high as 98% of MB and 80% of Cu^2+^ could be effectively removed by both adsorbents. At initial MB concentration of 400 mg/L, around 40% removal was obtained for SCB and SCBE. Even poorer performance of adsorption occurred on Cu^2+^, especially by SCBE. From Figure 5C, temperature had little influence on removal of MB and Cu^2+^ by SCB or SCBE. In any case, the removal rate for Cu^2+^ by SCBE remained lower than SCB, even though copper ions had two positive charges while MB had only one positive charge in solution. Adsorption mechanism depends not only on the surface groups but also on the zeta potential as well as other physicochemical properties. This demonstrates that different mechanisms might apply for adsorption of pollutants by SCB and SCBE. Some reports have observed a drastic decline in adsorption of MB by SCB along with the increasing temperature (Kerrou et al., 2021). These results suggest the potential of using SCB or SCBE in removal of dyes and metal ions from wastewater.

### 3.4 Adsorption of methylene blue and Cu^2+^ with sugarcane bagasse and SCBE: adsorption models

**FIGURE 6:**
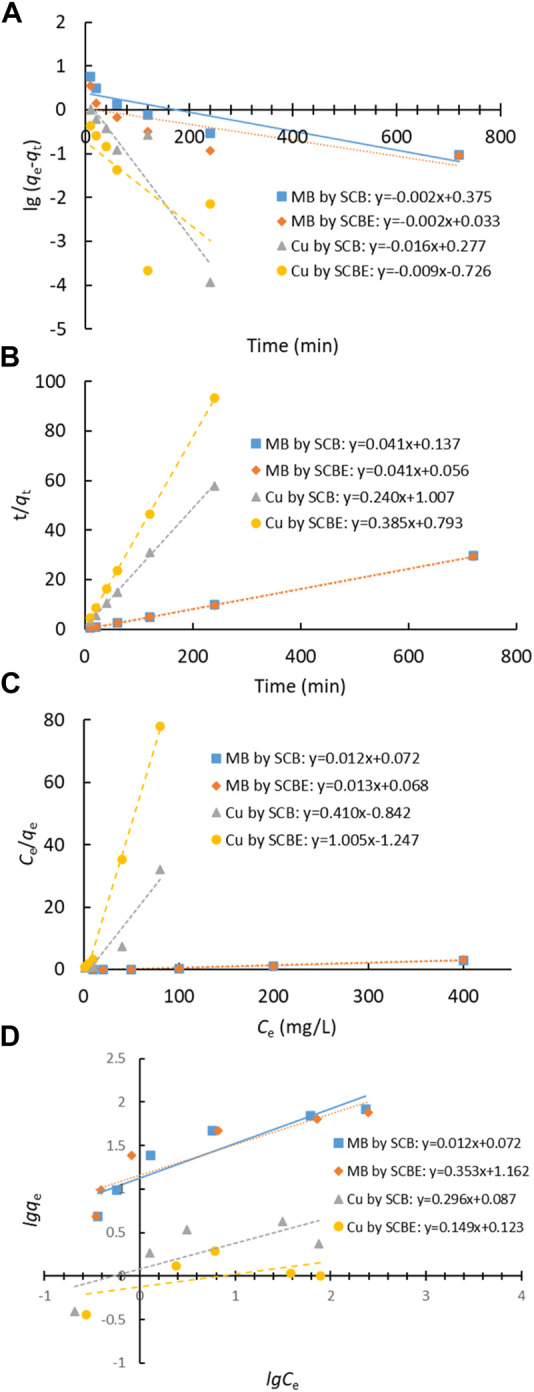
Adsorption models of dye MB and Cu^2+^ by SCB or SCBE: **(A)** Lagergren’s pseudo-first-order model, **(B)** Ho’s pseudo-second-order model, **(C)** Langmuir, and **(D)** Freundlich.

Adsorption data of contact time curve was fitted with Lagergren’s pseudo-first-order and Ho’s pseudo-second-order models (Figures 6A,B), and the model parameters are shown in Table 1. It was clear that the latter model better fitted the adsorption process in all the investigated cases (R^2^ > 0.999), implying that the enzymatic hydrolysis of SCB did not alter the adsorption kinetic feature. Moreover, the results also suggested that chemical interactions occurred between SCB or SCBE and pollutants *via* the exchange/sharing of electrons, where strong covalent bonds were formed (Han et al., 2010; Ezeonuegbu et al., 2021; Guan et al., 2022). Furthermore, the calculated *q*_e (cal)_ values and *q*_e (exp)_ obtained from experiment are very similar: 24.33 vs. 24.34 mg/g for MB by SCB, 24.57 vs. 24.63 mg/g for MB by SCBE, 4.17 vs. 4.14 mg/g for Cu^2+^ by SCB, and 2.59 vs. 2.58 mg/g for Cu^2+^ by SCBE. Kerrou et al. (2021) also confirmed the nature of pseudo-second-order kinetics of MB adsorption on SCB. Using citric acid–modified wheat straw (MWS) as an efficient adsorbent, the pseudo-second-order model has been found to perfectly describe the adsorption of Cu^2+^ and MB in the batch mode as well (Han et al., 2010).

**TABLE 1:** Kinetic parameters for MB dye and Cu^2+^ adsorption by SCB or SCBE.

Adsorption	Pseudo-first-order kinetic model			Pseudo-second-order kinetic model			Experimental
	*K*_1_ (min^−1^)	*q*_e (cal)_ (mg/g)	*R* ^*2*^	*K*_2_ (g/mg·min)	*q*_e (cal)_ (mg/g)	*R* ^*2*^	*q*_e (exp)_ (mg/g)
MB by SCB	0.0046	2.37	0.798	0.012	24.33	0.999	24.34
MB by SCBE	0.0046	1.08	0.614	0.023	24.57	0.999	24.63
Cu by SCB	0.036	1.89	0.872	0.057	4.17	0.999	4.137
Cu by SCBE	0.022	0.19	0.434	0.19	2.59	0.999	2.576

*q*_e (cal)_ is equilibrium adsorption capacity by calculation.

*q*_e (exp)_ is equilibrium adsorption capacity by experiment.

Langmuir and Freundlich isotherms were applied to fit the adsorption data (Figures 6C,D), and the corresponding parameters are listed in Table 2. From the correlation coefficient, Langmuir better described the adsorption process in the four cases, indicating the surface of SCB or SCBE were homogeneous and monolayer adsorption occurred on onto the adsorbents (Al-Mokhalelati et al., 2021). In this study, the interaction between adsorbates and adsorbents cannot be well explained by Freundlich because of the low R^2^ values (Liu et al., 2018). Compared with SCB, a reduced adsorption capacity was observed for SCBE: from 85.5 to 77.5 mg/g for MB and from 3.6 to 1.2 mg/g for Cu^2+^. From the separation factor *K*_*L*_, different results were obtained—the adsorption of MB was favorable (*K*_*L*_<1), whereas adsorption of Cu^2+^ was unfavorable due to the high values of *K*_*L*_, which was far higher than 1 (Kerrou et al., 2021). Generally, the bond energy of the adsorption reaction of SCBE with MB or metal ions was larger than that of SCB, as was reflected from the larger *K*_*L*_ values of SCBE (Karnitz et al., 2007). Even though enzymatic hydrolysis enlarged total pore volume and average pore size of SCBE, its adsorption capacity was reduced slightly. Varied adsorption capacities for MB (i.e., 9.4–90.1 mg/g) by different sources of original SCB have been reviewed in a recent review article (Aruna et al., 2021). In the meanwhile, comparison of pollutant removal by SCB/SCBE and representatives of modified/synthesized adsorbents are listed in Table 3. Reduction in adsorption capacity after biological modification (such as fungal pretreatment or colonization) has been reported in a few studies (Liu et al., 2018; Crespão et al., 2021), and the primary reason might be due to decreased cellulose content. Cellulose has been proved to be robust adsorbent for pollutants removal from water. This might explain why SCBE was outperformed by SCB regarding adsorption capacity because the latter had higher cellulose content. In addition, different adsorption mechanisms may apply to the interaction between adsorbents and adsorbates under different pH values, that is, chelation for Cu^2+^ (pH 4) and electrostatic interaction for MB (pH around 7.6), thus resulting in varied adsorption capacity (Li B. et al., 2019). Nonetheless, if compared with other agricultural wastes in raw form (e.g., rice/peanut husk or banana/orange peel), SCB as well as SCBE exhibit great adsorptive potential (Aruna et al., 2021).

**TABLE 2:** Isothermal parameters for MB dye and Cu^2+^ adsorption by SCB or SCBE.

Adsorption	Langmuir			Freundlich		
	*q*_m_ (mg/g)	*K*_*L*_	*R* ^*2*^	1/n	*K*_*F*_	*R* ^*2*^
MB by SCB	85.47	0.163	0.998	0.397	13.42	0.835
MB by SCBE	77.52	0.189	0.997	0.353	14.53	0.801
Cu by SCB	3.58	27.92	0.992	0.345	1.220	0.721
Cu by SCBE	1.18	28.26	0.995	0.173	0.750	0.382

**TABLE 3:** Comparison of MB dye and Cu^2+^ adsorption by SCB/SCBE and by some representatives of modified/synthesized adsorbents reported in the recent literature.

Adsorbent	MB		Cu^2+^		References
	Removal efficiency	Theoretical *q*_m_ (mg/g)	Removal efficiency	Theoretical *q*_m_ (mg/g)	
SCB/SCBE (with or without enzymatic hydrolysis)	98% by SCBE and 94% by SCB within 60 min (C_o_<100 mg/L)	SCB = 85.47 SCBE = 77.52	75% by SCBE and 80% by SCB within 60 min (C_o_<10 mg/L)	SCB = 3.58 SCBE = 1.18	This study
Modified sugarcane bagasse (MSBs) with succinic anhydride and polyamines	NI	NI	NM	MSB2 = 114; MSB5 = 139; MSB6 = 133.	Karnitz et al. (2007)
Charred xanthated sugarcane bagasse (CXSB)	NI	NI	NM	3.13 mol/kg dry sorbent	Homagai et al. (2010)
Succinylated-starch nanocrystals	NM	84	NM	84.07	Chen et al. (2019)
Polyaminocarboxylate-modified hydrochar	97% within 5 min (C_o_ < 500 mg/L)	1238.66	97% within 5 min (C_o_ < 100 mg/L)	140.65	Li et al. (2019a)
Biofoam adsorbent with nanocellulose (PUN_1_) and bamboo fiber (PUB_1_)	97% by PUN_1_ within 24 h (C_o_ = 25 mg/L)	PUN_1_ = 93.42	100% by PUB_1_ within 24 h (C_o_ = 15 mg/L)	PUB_1_ = 7.05	Qiu et al. (2021)
Fe_3_O_4_@SiO_2_@CS-TETA-GO	NM	529.1	NM	324.7	Wang et al. (2017)
Polymer adsorbent—SPCT	90% within 200 min (C_o_ = 50 mg/L)	952	95.4% within 200 min (C_o_ = 50 mg/L)	167	Cao et al. (2018)
Nano-hydroxyapatite (nHAP) immobilized with HA (humic acid)	100% within 8 h (C_o_ = 85 mg/L)	151.58	97.68% within 8 h (C_o_ = 25 mg/L)	85.32	Wei et al. (2020)

NI and NM means not investigated and not mentioned in the article, respectively.

Thermodynamics of adsorption of MB and Cu^2+^ was analyzed to reveal the underlying adsorption nature (Table 4). The negative values of ∆*G* ^o^ in all cases indicated the reaction would occur spontaneously. The positive Δ*H* ^o^ value identified an endothermic process for MB adsorption onto SCB or SCBE, while an exothermic reaction was suggested for Cu^2+^ adsorption by the two adsorbents (Δ*H* ^o^ < 0). In all cases, Δ*S* ^o^ >0 reflects an increased randomness at the solid/solution interface during the adsorption of MB or Cu^2+^ onto the two adsorbents. Kerrou et al. (2021) studied the adsorption of MB onto SCB and found ∆*G* ^o^, Δ*H* ^o^, and Δ*S* ^o^ of −4.35 kJ mol^−1^, −31.062 kJ mol^−1^, and −0.084 J·mol^−1^·K^−1^, respectively. These values were slightly different from our results, especially the values of Δ*H* ^o^ and Δ*S* ^o^ were opposite to the results in this study. In a research conducted by Han et al. (2010), adsorption of Cu^2+^ and MB by chemically modified wheat straw with citric acid (termed as MWS) was found to be spontaneous and endothermic.

**TABLE 4:** Thermodynamic parameters for MB dye and Cu^2+^ adsorption by SCB or SCBE.

Adsorption	Δ*G*°(J·mol^−1^)				Δ*H*° (J·mol^−1^)	Δ*S*° (J·mol^−1^·K^−1^)
	308 K	313 K	318 K	323 K		
MB by SCB	−2558.35	−2599.88	−2641.44	−2683.07	3.1505	8.3164
MB by SCBE	−2557.83	−2599.23	−2641.16	−2682.68	8.9874	8.3334
	298 K	308 K	318 K	328 K		
Cu by SCB	−2478	−2561.11	−2643.98	−2727.13	−3.8476	8.3026
Cu by SCBE	−2479.03	−2562.13	−2645.05	−2728.31	−3.3903	8.3075

## 4 Conclusion

Since many sugary energy crops such as sugarcane and sweet sorghum are widely grown worldwide, there is a great necessity to make full use of the co-produced bagasse. As demonstrated in this study, a considerable amount of residual soluble sugars is left in the bagasse after mechanically crushing for sugar juice extraction. These sugars are readily recovered by soaking in water and solid–liquid separation. The soluble sugar-free bagasse can be further hydrolyzed into fermentable sugars, for example, glucose, using cellulase enzyme. The combined recovered sugars can be potentially used as feedstock for biofuel production or other purposes. To enhance saccharification efficiency, potassium persulfate is a novel pretreatment reagent but the optimized condition should be investigated in the future. Both SCB before sugar recovery and SCBE after sugar recovery showed great capability as adsorbent for dye and metal ion removal from water. In comparison, SCBE exhibited slight reduction in adsorption capacity as a result of lower content of cellulose.

## Data Availability

The original contributions presented in the study are included in the article/Supplementary Material; further inquiries can be directed to the corresponding authors.
